# Supplementary material for: Machine Learning Accelerates Crystallization for Structure Determination
Source: Angew Chem Int Ed Engl. 2026 May 4;65(25):e1218503. doi: 10.1002/anie.1218503 (PMC13266958; doi:10.1002/anie.1218503)

checkCIF/PLATON report

Structure factors have been supplied for datablock(s) lcz114

THIS REPORT IS FOR GUIDANCE ONLY. IF USED AS PART OF A REVIEW PROCEDURE FOR PUBLICATION, IT SHOULD NOT REPLACE THE EXPERTISE OF AN EXPERIENCED CRYSTALLOGRAPHIC REFEREE.

No syntax errors found. CIF dictionary Interpreting this report

Datablock: lcz114

Bond precision:	C-C = 0.0356 A		Wavelength=1.54184
Cell:	a=13.5064(2)	b=31.9763(5)	c=24.8145(4)
	alpha=90	beta=94.634(1)	gamma=90
Temperature:	100 K		

	Calculated	Reported
Volume	10682.0(3)	10682.0(3)
Space group	P 21	P 1 21 1
Hall group	P 2yb	P 2yb
Moiety formula	C145 H157 Ag9 F54 N18 O28, C15 H3 Ag3 F18 N6 [+ solvent]	C50 H74 O14, 2(C15 H3 Ag3 F18 N6)
Sum formula	C160 H160 Ag12 F72 N24 O28 [+ solvent]	C80 H80 Ag6 F36 N12 O14
Mr	5529.53	2764.78
Dx,g cm-3	1.719	1.719
Z	2	4
Mu (mm-1)	9.762	9.762
F000	5448.0	5448.0
F000'	5472.19	
h,k,lmax	17,40,31	17,40,30
Nref	45655[23250]	33573
Tmin,Tmax	0.150,0.129	0.611,1.000
Tmin'	0.042	

Correction method= # Reported T Limits: Tmin=0.611 Tmax=1.000
AbsCorr = MULTI-SCAN

Data completeness= 1.44/0.74 Theta(max)= 77.957

R(reflections)= 0.0896(26842) wR2(reflections)= 0.2595(33573)

S = 1.021 Npar= 2579

The following ALERTS were generated. Each ALERT has the format
test-name_ALERT_alert-type_alert-level.
Click on the hyperlinks for more details of the test.

Alert level B

PLAT342_ALERT_3_B Low Bond Precision on C-C Bonds .... 0.03563 Ang.

Author Response: Disordered structure.

PLAT412_ALERT_2_B Short Intra XH3 .. XHn H11B ..H63C . 1.76 Ang.
x,y,z = 1_555 Check

Author Response: This Alert is due to close contacts between protons of highly disordered terminal methyl groups with high thermal parameters.

PLAT420_ALERT_2_B D-H Bond Without Acceptor O00M --H00M . Please Check

Author Response: The solvent molecules are highly disordered, which supposed to form hydrogen bond with O have been removed by the SQUEEZE routine in the PLATON software package.

PLAT780_ALERT_1_B Coordinates do not Form a Properly Connected Set Please Do !

Author Response: The alert is due to a large number of co-crystallized molecules in the which sometimes do not show as a connected set. This is acceptable from a crystallographic point of view.

PLAT973_ALERT_2_B Check Calcd Positive Resid. Density on Ag01 1.74 eA-3

Author Response: This Alert is due to presence of residual density in the presence of heavy metal atom (Ag)

```
PLAT973_ALERT_2_B Check Calcd Positive Resid. Density on      Ag02       1.61 eA-3
```

Author Response: This Alert is due to presence of residual density in the presence of heavy metal atom (Ag)

Alert level C

```
PLAT084_ALERT_3_C High wR2 Value (i.e. > 0.25) ...................       0.26 Report
PLAT213_ALERT_2_C Atom F015            has ADP max/min Ratio .....        3.5 prolat
PLAT213_ALERT_2_C Atom C079            has ADP max/min Ratio .....        3.1 prolat
PLAT220_ALERT_2_C NonSolvent   Resd 1  C   Ueq(max)/Ueq(min) Range        4.5 Ratio
PLAT220_ALERT_2_C NonSolvent   Resd 1  F   Ueq(max)/Ueq(min) Range        4.3 Ratio
PLAT220_ALERT_2_C NonSolvent   Resd 1  N   Ueq(max)/Ueq(min) Range        3.3 Ratio
PLAT222_ALERT_3_C NonSolvent Resd 1  H   Uiso(max)/Uiso(min) Range        5.2 Ratio
PLAT241_ALERT_2_C High   'MainMol' Ueq as Compared to Neighbors of       O01T Check
PLAT241_ALERT_2_C High   'MainMol' Ueq as Compared to Neighbors of       O03A Check
PLAT241_ALERT_2_C High   'MainMol' Ueq as Compared to Neighbors of       O03N Check
PLAT241_ALERT_2_C High   'MainMol' Ueq as Compared to Neighbors of       O01W Check
PLAT241_ALERT_2_C High   'MainMol' Ueq as Compared to Neighbors of          C Check
PLAT241_ALERT_2_C High   'MainMol' Ueq as Compared to Neighbors of         C3 Check
PLAT241_ALERT_2_C High   'MainMol' Ueq as Compared to Neighbors of       C03D Check
PLAT241_ALERT_2_C High   'MainMol' Ueq as Compared to Neighbors of        C11 Check
PLAT241_ALERT_2_C High   'MainMol' Ueq as Compared to Neighbors of       C05W Check
PLAT241_ALERT_2_C High   'MainMol' Ueq as Compared to Neighbors of        C28 Check
PLAT241_ALERT_2_C High   'MainMol' Ueq as Compared to Neighbors of       C04D Check
PLAT241_ALERT_2_C High   'MainMol' Ueq as Compared to Neighbors of       C05O Check
PLAT241_ALERT_2_C High   'MainMol' Ueq as Compared to Neighbors of       C06O Check
PLAT241_ALERT_2_C High   'MainMol' Ueq as Compared to Neighbors of       C067 Check
PLAT241_ALERT_2_C High   'MainMol' Ueq as Compared to Neighbors of       C104 Check
PLAT241_ALERT_2_C High   'MainMol' Ueq as Compared to Neighbors of       C07J Check
PLAT241_ALERT_2_C High   'MainMol' Ueq as Compared to Neighbors of       C04T Check
PLAT241_ALERT_2_C High   'MainMol' Ueq as Compared to Neighbors of       C05U Check
PLAT241_ALERT_2_C High   'MainMol' Ueq as Compared to Neighbors of       C666 Check
PLAT241_ALERT_2_C High   'MainMol' Ueq as Compared to Neighbors of       N05J Check
PLAT241_ALERT_2_C High   'MainMol' Ueq as Compared to Neighbors of       C07L Check
PLAT242_ALERT_2_C Low    'MainMol' Ueq as Compared to Neighbors of         C4 Check
PLAT242_ALERT_2_C Low    'MainMol' Ueq as Compared to Neighbors of       C02S Check
PLAT242_ALERT_2_C Low    'MainMol' Ueq as Compared to Neighbors of       C02Y Check
PLAT242_ALERT_2_C Low    'MainMol' Ueq as Compared to Neighbors of       C03R Check
PLAT242_ALERT_2_C Low    'MainMol' Ueq as Compared to Neighbors of       C06S Check
PLAT242_ALERT_2_C Low    'MainMol' Ueq as Compared to Neighbors of        C33 Check
PLAT242_ALERT_2_C Low    'MainMol' Ueq as Compared to Neighbors of       C07V Check
PLAT242_ALERT_2_C Low    'MainMol' Ueq as Compared to Neighbors of       C052 Check
PLAT242_ALERT_2_C Low    'MainMol' Ueq as Compared to Neighbors of        C45 Check
PLAT242_ALERT_2_C Low    'MainMol' Ueq as Compared to Neighbors of       C075 Check
PLAT242_ALERT_2_C Low    'MainMol' Ueq as Compared to Neighbors of       C01X Check
PLAT242_ALERT_2_C Low    'MainMol' Ueq as Compared to Neighbors of       C03T Check
PLAT242_ALERT_2_C Low    'MainMol' Ueq as Compared to Neighbors of       C05C Check
PLAT242_ALERT_2_C Low    'MainMol' Ueq as Compared to Neighbors of       C06A Check
PLAT242_ALERT_2_C Low    'MainMol' Ueq as Compared to Neighbors of       C042 Check
PLAT242_ALERT_2_C Low    'MainMol' Ueq as Compared to Neighbors of       C02T Check
PLAT242_ALERT_2_C Low    'MainMol' Ueq as Compared to Neighbors of       C03G Check
PLAT242_ALERT_2_C Low    'MainMol' Ueq as Compared to Neighbors of       N07A Check
PLAT260_ALERT_2_C Large Average Ueq of Residue Including      Ag01      0.101 Check
```

```
PLAT260_ALERT_2_C Large Average Ueq of Residue Including      Ag0A      0.122 Check
PLAT360_ALERT_2_C Short  C(sp3)-C(sp3) Bond  C11      - C052     .       1.43 Ang.
PLAT360_ALERT_2_C Short  C(sp3)-C(sp3) Bond  C01X     - C03F     .       1.34 Ang.
PLAT360_ALERT_2_C Short  C(sp3)-C(sp3) Bond  C02V     - C042     .       1.41 Ang.
PLAT360_ALERT_2_C Short  C(sp3)-C(sp3) Bond  C03J     - C049     .       1.43 Ang.
PLAT360_ALERT_2_C Short  C(sp3)-C(sp3) Bond  C03O     - C05O     .       1.43 Ang.
PLAT360_ALERT_2_C Short  C(sp3)-C(sp3) Bond  C05H     - C041     .       1.43 Ang.
PLAT361_ALERT_2_C Long   C(sp3)-C(sp3) Bond  C3       - C07V     .       1.65 Ang.
PLAT361_ALERT_2_C Long   C(sp3)-C(sp3) Bond  C07G     - C07N     .       1.65 Ang.
PLAT361_ALERT_2_C Long   C(sp3)-C(sp3) Bond  C035     - C056     .       1.65 Ang.
PLAT362_ALERT_2_C Short  C(sp3)-C(sp2) Bond  C04Z     - C06F     .       1.37 Ang.
PLAT362_ALERT_2_C Short  C(sp3)-C(sp2) Bond  C07J     - C077     .       1.40 Ang.
PLAT362_ALERT_2_C Short  C(sp3)-C(sp2) Bond  C42      - C07L     .       1.40 Ang.
PLAT362_ALERT_2_C Short  C(sp3)-C(sp2) Bond  C07Q     - C07Y     .       1.37 Ang.
PLAT363_ALERT_2_C Long   C(sp3)-C(sp2) Bond  C06R     - C06S     .       1.63 Ang.
PLAT368_ALERT_2_C Short  C(sp2)-C(sp2) Bond  C04Q     - C064     .       1.23 Ang.
PLAT368_ALERT_2_C Short  C(sp2)-C(sp2) Bond  C06D     - C06S     .       1.18 Ang.
PLAT369_ALERT_2_C Long   C(sp2)-C(sp2) Bond  C05N     - C07J     .       1.53 Ang.
PLAT416_ALERT_2_C Short Intra D-H..H-D       H00K     ..H00N     .       1.96 Ang.
                                                      x,y,z  =      1_555 Check
PLAT911_ALERT_3_C Missing FCF Refl Between Thmin & STh/L=    0.600        71 Report
                5  0  0,   1  4  0,   1  6  0,   2 12  0,   9 15  0, -15  0  1,
               -5  0  1,   2  0  1,  -4  1  1,   4  1  1,  -1  3  1,   0  3  1,
               -9 15  1,   1 26  1,   0 37  1,  -8  0  2,  -5  0  2,  -1  0  2,
                0  0  2,   1  0  2,  -1  3  2,   0  5  2,  -6  6  2,  -3 11  2,
               -1  0  3,   0  0  3,   0  2  3,  -1  4  3,  -9  8  3,  -4  0  4,
                    (   41 More Missing: see the .ckf listing file)
PLAT915_ALERT_3_C No Flack x Check Done: Low Friedel Pair Coverage         50 %
PLAT971_ALERT_2_C Check Calcd Resid. Dens.  0.76Ang From Ag04         1.91 eA-3
PLAT971_ALERT_2_C Check Calcd Resid. Dens.  0.78Ang From Ag08         1.82 eA-3
PLAT971_ALERT_2_C Check Calcd Resid. Dens.  0.21Ang From Ag03         1.61 eA-3
PLAT971_ALERT_2_C Check Calcd Resid. Dens.  0.78Ang From Ag06         1.59 eA-3
PLAT971_ALERT_2_C Check Calcd Resid. Dens.  0.86Ang From Ag07         1.58 eA-3
PLAT971_ALERT_2_C Check Calcd Resid. Dens.  0.76Ang From Ag07         1.57 eA-3
PLAT972_ALERT_2_C Check Calcd Resid. Dens.  0.54Ang From Ag09        -2.08 eA-3
PLAT972_ALERT_2_C Check Calcd Resid. Dens.  0.61Ang From Ag0A        -1.91 eA-3
PLAT972_ALERT_2_C Check Calcd Resid. Dens.  0.52Ang From Ag09        -1.91 eA-3
PLAT972_ALERT_2_C Check Calcd Resid. Dens.  0.50Ang From Ag0A        -1.85 eA-3
PLAT972_ALERT_2_C Check Calcd Resid. Dens.  0.40Ang From Ag0A        -1.84 eA-3
PLAT972_ALERT_2_C Check Calcd Resid. Dens.  0.48Ang From Ag0B        -1.81 eA-3
PLAT972_ALERT_2_C Check Calcd Resid. Dens.  0.61Ang From Ag09        -1.78 eA-3
PLAT972_ALERT_2_C Check Calcd Resid. Dens.  0.36Ang From Ag0A        -1.76 eA-3
PLAT972_ALERT_2_C Check Calcd Resid. Dens.  0.35Ang From Ag0B        -1.75 eA-3
PLAT972_ALERT_2_C Check Calcd Resid. Dens.  0.56Ang From Ag0B        -1.74 eA-3
PLAT973_ALERT_2_C Check Calcd Positive Resid. Density on      Ag04      1.29 eA-3
```

Author Response: This Alert is due to presence of residual density in the presence of heavy metal atom (Ag)

```
PLAT973_ALERT_2_C Check Calcd Positive Resid. Density on      Ag08      1.22 eA-3
```

Author Response: This Alert is due to presence of residual density in the presence of heavy metal atom (Ag)

```
PLAT977_ALERT_2_C Check Negative Difference Density on H05X      .      -0.39 eA-3
PLAT977_ALERT_2_C Check Negative Difference Density on H35B      .      -0.42 eA-3
PLAT977_ALERT_2_C Check Negative Difference Density on H05F      .      -0.53 eA-3
PLAT977_ALERT_2_C Check Negative Difference Density on H05I      .      -0.35 eA-3
PLAT977_ALERT_2_C Check Negative Difference Density on H99B      .      -0.37 eA-3
```

Alert level G

```
PLAT002_ALERT_2_G Number of Distance or Angle Restraints on AtSite         53 Note
PLAT003_ALERT_2_G Number of Uiso or U(i,j)  Restrained non-H-Atoms         96 Report
PLAT004_ALERT_5_G Polymeric Structure Found with Maximum Dimension          1 Info
PLAT007_ALERT_5_G Number of Unrefined Donor-H Atoms .............           6 Report
              H00K  H00P  H01O  H00J  H00M  H00N
PLAT033_ALERT_4_G Flack x Value Deviates > 3.0 * Sigma from Zero .      0.121 Note
PLAT041_ALERT_1_G Calc. and Reported SumFormula    Strings  Differ     Please Check
              Calc: C160 H160 Ag12 F72 N24 O28
              Rep.: C80 H80 Ag6 F36 N12 O14
PLAT042_ALERT_1_G Calc. and Reported MoietyFormula Strings  Differ     Please Check
              Calc: C145 H157 Ag9 F54 N18 O28, C15 H3 Ag3 F18 N6
              Rep.: C50 H74 O14, 2(C15 H3 Ag3 F18 N6)
PLAT045_ALERT_1_G Calculated and Reported Z Differ by a Factor ...      0.500 Check
PLAT072_ALERT_2_G SHELXL First  Parameter in WGHT  Unusually Large       0.20 Report
PLAT171_ALERT_4_G The CIF-Embedded .res File Contains EADP Records         11 Report
PLAT172_ALERT_4_G The CIF-Embedded .res File Contains DFIX Records         50 Report
PLAT174_ALERT_4_G The CIF-Embedded .res File Contains FLAT Records          2 Report
PLAT177_ALERT_4_G The CIF-Embedded .res File Contains DELU Records         17 Report
PLAT178_ALERT_4_G The CIF-Embedded .res File Contains SIMU Records         23 Report
PLAT186_ALERT_4_G The CIF-Embedded .res File Contains ISOR Records         26 Report
PLAT188_ALERT_3_G A Non-default SIMU Restraint Value has been used     0.0200 Report
PLAT188_ALERT_3_G A Non-default SIMU Restraint Value has been used     0.0200 Report
PLAT188_ALERT_3_G A Non-default SIMU Restraint Value has been used     0.0200 Report
PLAT242_ALERT_2_G Low    'MainMol' Ueq as Compared to Neighbors of         C2 Check
PLAT242_ALERT_2_G Low    'MainMol' Ueq as Compared to Neighbors of       C02W Check
PLAT242_ALERT_2_G Low    'MainMol' Ueq as Compared to Neighbors of       C04B Check
PLAT242_ALERT_2_G Low    'MainMol' Ueq as Compared to Neighbors of       C04K Check
PLAT242_ALERT_2_G Low    'MainMol' Ueq as Compared to Neighbors of        C15 Check
PLAT242_ALERT_2_G Low    'MainMol' Ueq as Compared to Neighbors of       C04P Check
PLAT242_ALERT_2_G Low    'MainMol' Ueq as Compared to Neighbors of        C20 Check
PLAT242_ALERT_2_G Low    'MainMol' Ueq as Compared to Neighbors of       C048 Check
PLAT242_ALERT_2_G Low    'MainMol' Ueq as Compared to Neighbors of       C04Z Check
PLAT242_ALERT_2_G Low    'MainMol' Ueq as Compared to Neighbors of       C06U Check
PLAT242_ALERT_2_G Low    'MainMol' Ueq as Compared to Neighbors of       C077 Check
PLAT242_ALERT_2_G Low    'MainMol' Ueq as Compared to Neighbors of       C02X Check
PLAT242_ALERT_2_G Low    'MainMol' Ueq as Compared to Neighbors of       C02Z Check
PLAT242_ALERT_2_G Low    'MainMol' Ueq as Compared to Neighbors of       C031 Check
PLAT242_ALERT_2_G Low    'MainMol' Ueq as Compared to Neighbors of       C032 Check
PLAT242_ALERT_2_G Low    'MainMol' Ueq as Compared to Neighbors of       C046 Check
PLAT242_ALERT_2_G Low    'MainMol' Ueq as Compared to Neighbors of         C1 Check
PLAT242_ALERT_2_G Low    'MainMol' Ueq as Compared to Neighbors of        C42 Check
PLAT242_ALERT_2_G Low    'MainMol' Ueq as Compared to Neighbors of       C05I Check
PLAT242_ALERT_2_G Low    'MainMol' Ueq as Compared to Neighbors of       C06E Check
PLAT242_ALERT_2_G Low    'MainMol' Ueq as Compared to Neighbors of       C07Y Check
PLAT242_ALERT_2_G Low    'MainMol' Ueq as Compared to Neighbors of       C063 Check
PLAT303_ALERT_2_G Full Occupancy Atom H01O      with # Connections       2.00 Check
PLAT303_ALERT_2_G Full Occupancy Atom H00J      with # Connections       2.00 Check
PLAT303_ALERT_2_G Full Occupancy Atom H00N      with # Connections       2.00 Check
PLAT343_ALERT_2_G Unusual sp3      Angle Range in Main Residue for         C2 Check
```

PLAT343_ALERT_2_G Unusual sp3 Angle Range in Main Residue for C15 Check
PLAT343_ALERT_2_G Unusual sp3 Angle Range in Main Residue for C33 Check
PLAT343_ALERT_2_G Unusual sp3 Angle Range in Main Residue for C35 Check
PLAT343_ALERT_2_G Unusual sp3 Angle Range in Main Residue for C07V Check
PLAT432_ALERT_2_G Short Inter X...Y Contact N060 ..C07X . 3.02 Ang.
x,y,z = 1_555 Check
PLAT432_ALERT_2_G Short Inter X...Y Contact N069 ..C07X . 3.01 Ang.
x,y,z = 1_555 Check
PLAT434_ALERT_2_G Short Inter HL..HL Contact F2 ..F04N . 2.64 Ang.
-1+x,y,z = 1_455 Check
PLAT434_ALERT_2_G Short Inter HL..HL Contact F02H ..F02O . 2.76 Ang.
2-x,-1/2+y,1-z = 2_746 Check
PLAT434_ALERT_2_G Short Inter HL..HL Contact F04M ..F02O . 2.84 Ang.
2-x,-1/2+y,1-z = 2_746 Check
PLAT434_ALERT_2_G Short Inter HL..HL Contact F05E ..F02I . 2.82 Ang.
1-x,-1/2+y,1-z = 2_646 Check
PLAT434_ALERT_2_G Short Inter HL..HL Contact F065 ..F029 . 2.84 Ang.
2-x,-1/2+y,1-z = 2_746 Check
PLAT606_ALERT_4_G Solvent Accessible VOID(S) in Crystal Structure ! Info
PLAT720_ALERT_4_G Number of Unusual/Non-Standard Labels .... 391 Note

Ha	Hb	F00D	F00G	F00H	F00L	F00Q	F00V
O00K	H00K	O00P	H00P	N00R	N00U	N00Y	Ag01
F01I	F01S	O01J	O01O	H01O	O01T	O01U	O01Z
N01E	N01K	N01M	C01N	C01Q	H01Q	C01R	Ag02
O02M	C02L	H02L	C02S	C02W	C02Y	Ag03	F03C
F03E	O03A	O03K	O03N	C03D	H03A	H03B	C03Q
H03Q	C03R	C03S	H03S	C03U	H03U	F04H	F04U
O04V	C04B	C04K	C04P	C04Q	H04Q	C04X	H04A
H04B	H04C	F05A	C05B	C05D	C05F	C05L	H05L
C05R	H05R	C05W	H05Y	C05X	H05X	C05Z	H05A
H05B	F06Y	C06D	H06D	C06H	H06H	C06R	H06R
C06S	C06Z	H06A	H06B	H06C	C07G	H07A	H07B
H07C	C07I	H07I	C07N	H07N	C07U	H07D	H07E
H07F	C07V	H07V	C07X	H07X	F011	O013	O018
F020	F022	F025	C034	C035	H035	C036	C039
C040	H040	C048	C052	H052	C054	H054	C055
H055	C056	H05C	H05D	H05E	C062	H06E	H06F
C064	H064	C066	H06G	H06I	H06J	C073	H073
C075	C079	H079	O00E	O00F	O00I	O00J	H00J
O00M	H00M	O00N	H00N	O00O	O00S	O00W	O00Z
O01A	O01W	C01B	C01D	C01P	H01A	H01B	C01V
H01V	C01X	H01X	C01Y	H01C	H01D	C02A	H02A
C02D	H02B	H02C	C02K	H02K	C02P	H02P	C02Q
H02Q	C02R	H02R	C02U	H02U	C02V	H02V	C03F
H03F	C03H	H03H	C03I	H03I	C03J	H03J	C03O
H03O	C03P	C03T	H03C	H03D	C03W	C03Y	H03E
H03G	H03K	C04C	H04D	C04D	H04E	H04F	C04E
H04G	C05C	H05F	C05H	H05G	H05H	H05I	C05M
H05J	H05K	C05O	H05M	H05N	C05S	C05T	H05O
H05P	H05Q	C05Y	H05S	H05T	C06A	H06K	H06L
C06B	H06M	C06O	H06N	H06O	C06P	H06P	H06Q
H06S	C06Q	H06T	H06U	H06V	O012	C021	H021
C023	C028	H028	C041	H041	C042	H042	C045
H045	C049	H049	C051	H051	C059	H05U	H05V
H05W	C067	H06W	H06X	C070	H07G	H07H	H07J
C072	H07K	H07L	H07M	Ag0A	Ag0B	F02H	F03Z
F04L	F04M	F04N	F04Y	N04R	F05E	F05K	F05P

```
             F05V    N05Q    C05I    F06L    F06V    C06E    N07A    C07L
             C06W    H06Y    C07O    N05J    C07F    C07M    C07Q    C053
             H053    C06J    N060    N069    C07Y    Ag09    F037    F038
             F050    F058    C063    F065    Ag0C    F02C    F02F    F02J
             F03B    F03L    F04G    N04W    C04Z    C05N    H05Z    F06I
             C06F    C06U    Ag07    F07H    C07J    Ag08    F024    N033
             C077    C06M    N00T    N00X    F01G    F01L    N01C    N01F
             F02E    F02I    F02O    N02B    C02N    C02T    C02X    C02Z
             F03V    C03G    C03M    C03X    H03X    Ag04    F04A    F04F
             C04O    C04S    H04S    C04T    H04T    Ag05    C05U    Ag06
             F010    N014    F015    F016    F017    F019    F026    F027
             F029    C030    C031    C032    C046    F047    F057
PLAT721_ALERT_1_G Bond    Calc     0.97000, Rep     0.96000 Dev...       0.01 Ang.
             C06Z     -H06C           1_555   1_555 ........     #  128 Check
PLAT779_ALERT_4_G Suspect or Irrelevant (Bond) Angle(s) in CIF ...      37.40 Deg.
             C15  -F5   -F6      1_555   1_555   1_555 ......     #  745 Check
PLAT802_ALERT_4_G CIF Input Record(s) with more than 80 Characters          1 Info
PLAT860_ALERT_3_G Number of Least-Squares Restraints ............         733 Note
PLAT868_ALERT_4_G ALERTS Due to the Use of _smtbx_masks Suppressed          ! Info
PLAT910_ALERT_3_G Missing FCF Reflection(s) Below Theta(Min)[Deg]=       3.29 Note
               0  2  0,   0  0  1,   0  1  1,
PLAT912_ALERT_4_G Missing # of FCF Reflections Above STh/L=  0.600        840 Note
PLAT933_ALERT_2_G Number of HKL-OMIT Records in Embedded .res File         77 Note
              -6 -6  2,  -6  0  6,  -6  6  2,  -5-12  1,  -5  0  1,  -5  0  2,
              -4 -4  7,  -4 -1  1,  -4  0  4,  -4  1  1,  -4  2  3,  -3-11  2,
              -3 -9  4,  -3  9  4,  -2-16  1,  -2 -6  1,  -2  3  3,  -1 -4  1,
              -1 -3  1,  -1  0  2,  -1  0  3,  -1  3  1,  -1  7  7,  -1 10  2,
               0-37  1,   0 -5 -5,   0 -2  3,   0  0  2,   0  0  3,   0  2  3,
               0  3  1,   0  5  2,   0  5  5,   1-11  2,   1 -7 10,   1 -6  0,
               1 -5  4,   1 -4  0,   1 -1  3,   1  0  2,   1  3  2,   1  4  0,
               1  4  2,   1  4  4,   1  4  6,   2-12  0,   2  0  1,   2  0  8,
               2  0 11,   2  0 13,
PLAT941_ALERT_3_G Average HKL Measurement Multiplicity ..........         2.7 Low
PLAT969_ALERT_5_G The 'Henn et al.' R-Factor-gap value ..........      3.554 Note
             Predicted wR2: Based on SigI**2  7.30 or SHELX Weight 25.41
PLAT978_ALERT_2_G Number C-C Bonds with Positive Residual Density.          0 Info
PLAT992_ALERT_5_G Repd & Actual _reflns_number_gt Values Differ by          2 Check
```

```
   0 ALERT level A = Most likely a serious problem - resolve or explain
   6 ALERT level B = A potentially serious problem, consider carefully
  91 ALERT level C = Check. Ensure it is not caused by an omission or oversight
  69 ALERT level G = General information/check it is not something unexpected

   5 ALERT type 1 CIF construction/syntax error, inconsistent or missing data
 133 ALERT type 2 Indicator that the structure model may be wrong or deficient
  11 ALERT type 3 Indicator that the structure quality may be low
  13 ALERT type 4 Improvement, methodology, query or suggestion
   4 ALERT type 5 Informative message, check
```

Validation response form

Please find below a validation response form (VRF) that can be filled in and pasted into your CIF.

```
# start Validation Reply Form
_vrf_PLAT084_lcz114
;
PROBLEM: High wR2 Value (i.e. > 0.25) ...................       0.26 Report
RESPONSE: ...
;
_vrf_PLAT213_lcz114
;
PROBLEM: Atom F015            has ADP max/min Ratio .....        3.5 prolat
RESPONSE: ...
;
_vrf_PLAT220_lcz114
;
PROBLEM: NonSolvent   Resd 1  C   Ueq(max)/Ueq(min) Range        4.5 Ratio
RESPONSE: ...
;
_vrf_PLAT222_lcz114
;
PROBLEM: NonSolvent Resd 1  H   Uiso(max)/Uiso(min) Range        5.2 Ratio
RESPONSE: ...
;
_vrf_PLAT241_lcz114
;
PROBLEM: High   'MainMol' Ueq as Compared to Neighbors of       O01T Check
RESPONSE: ...
;
_vrf_PLAT242_lcz114
;
PROBLEM: Low    'MainMol' Ueq as Compared to Neighbors of         C4 Check
RESPONSE: ...
;
_vrf_PLAT260_lcz114
;
PROBLEM: Large Average Ueq of Residue Including      Ag01      0.101 Check
RESPONSE: ...
;
_vrf_PLAT360_lcz114
;
PROBLEM: Short  C(sp3)-C(sp3) Bond  C11      - C052     .       1.43 Ang.
RESPONSE: ...
;
_vrf_PLAT361_lcz114
;
PROBLEM: Long   C(sp3)-C(sp3) Bond  C3       - C07V     .       1.65 Ang.
RESPONSE: ...
;
_vrf_PLAT362_lcz114
;
PROBLEM: Short  C(sp3)-C(sp2) Bond  C04Z     - C06F     .       1.37 Ang.
RESPONSE: ...
;
_vrf_PLAT363_lcz114
;
PROBLEM: Long   C(sp3)-C(sp2) Bond  C06R     - C06S     .       1.63 Ang.
RESPONSE: ...
;
_vrf_PLAT368_lcz114
```

```
;
PROBLEM: Short  C(sp2)-C(sp2) Bond  C04Q     - C064     .       1.23 Ang.
RESPONSE: ...
;
_vrf_PLAT369_lcz114
;
PROBLEM: Long   C(sp2)-C(sp2) Bond  C05N     - C07J     .       1.53 Ang.
RESPONSE: ...
;
_vrf_PLAT416_lcz114
;
PROBLEM: Short Intra D-H..H-D       H00K     ..H00N     .       1.96 Ang.
RESPONSE: ...
;
_vrf_PLAT911_lcz114
;
PROBLEM: Missing FCF Refl Between Thmin & STh/L=    0.600         71 Report
RESPONSE: ...
;
_vrf_PLAT915_lcz114
;
PROBLEM: No Flack x Check Done: Low Friedel Pair Coverage         50 %
RESPONSE: ...
;
_vrf_PLAT971_lcz114
;
PROBLEM: Check Calcd Resid. Dens.  0.76Ang From Ag04            1.91 eA-3
RESPONSE: ...
;
_vrf_PLAT972_lcz114
;
PROBLEM: Check Calcd Resid. Dens.  0.54Ang From Ag09           -2.08 eA-3
RESPONSE: ...
;
_vrf_PLAT977_lcz114
;
PROBLEM: Check Negative Difference Density on H05X      .      -0.39 eA-3
RESPONSE: ...
;
# end Validation Reply Form
```

It is advisable to attempt to resolve as many as possible of the alerts in all categories. Often the minor alerts point to easily fixed oversights, errors and omissions in your CIF or refinement strategy, so attention to these fine details can be worthwhile. It is up to the individual to critically assess their own results and, if necessary, seek expert advice.

PLATON version of 17/09/2025; check.def file version of 17/09/2025

---

duplicate check

No duplication found

Datablock lcz114 - ellipsoid plot

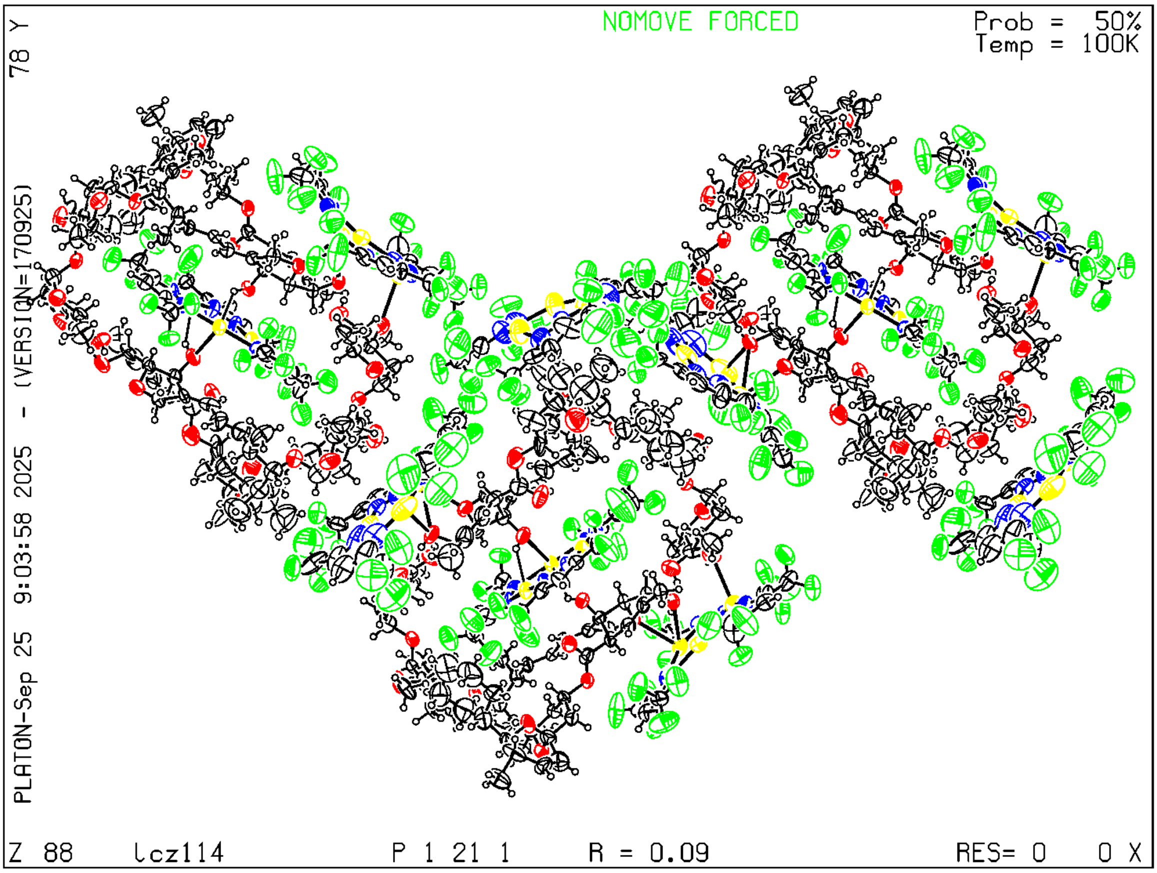

Supplement: Supplementary file 2 — Supporting File 2: anie72462‐sup‐0002‐DataFile.zip. [file ANIE-65-e1218503-s002.zip › supplementary_materials_crystallographic_data submit.zip!/cif_and_checkcif(1-114)/Ag3Pz3_114/Ag3Pz3_114_checkcif.pdf]
